# Functional Anatomy of the Trimer Apex Reveals Key Hydrophobic Constraints That Maintain the HIV-1 Envelope Spike in a Closed State

**DOI:** 10.1128/mBio.00090-21

**Published:** 2021-03-30

**Authors:** Peng Zhang, Alice L. Kwon, Christina Guzzo, Qingbo Liu, Hana Schmeisser, Huiyi Miao, Yin Lin, Raffaello Cimbro, Jinghe Huang, Mark Connors, Stephen D. Schmidt, Michael A. Dolan, Anthony A. Armstrong, Paolo Lusso

**Affiliations:** a Laboratory of Immunoregulation, National Institute of Allergy and Infectious Diseases, NIH, Bethesda, Maryland, USA; b Division of Rheumatology, Johns Hopkins University School of Medicine, Baltimore, Maryland, USA; c Vaccine Research Center, National Institute of Allergy and Infectious Diseases, NIH, Bethesda, Maryland, USA; d Bioinformatics and Computational Biosciences Branch, Office of Cyber Infrastructure and Computational Biology, National Institute of Allergy and Infectious Diseases, NIH, Bethesda, Maryland, USA; Harvard University; University of KwaZulu-Natal

**Keywords:** HIV-1, envelope, trimer, prefusion state, neutralizing antibodies, immune evasion, envelope glycoprotein, protein structure-function

## Abstract

The human immunodeficiency virus type 1 (HIV-1) envelope trimer maintains a closed, metastable configuration to protect vulnerable epitopes from neutralizing antibodies. Here, we identify key hydrophobic constraints at the trimer apex that function as global stabilizers of the HIV-1 envelope spike configuration. Mutation of individual residues within four hydrophobic clusters that fasten together the V1V2, V3, and C4 domains at the apex of gp120 dramatically increases HIV-1 sensitivity to weak and restricted neutralizing antibodies targeting epitopes that are largely concealed in the prefusion Env spike, consistent with the adoption of a partially open trimer configuration. Conversely, the same mutations decrease the sensitivity to broad and potent neutralizing antibodies that preferentially recognize the closed trimer. Sera from chronically HIV-infected patients neutralize open mutants with enhanced potency, compared to the wild-type virus, suggesting that a large fraction of host-generated antibodies target concealed epitopes. The identification of structural constraints that maintain the HIV-1 envelope in an antibody-protected state may inform the design of a protective vaccine.

## Abstract (precis)

Elucidating the structure and function of the HIV-1 outer envelope proteins is critical for the design of an effective vaccine. Despite the availability of many high-resolution structures, key functional correlates in the envelope trimer remain undefined.

## INTRODUCTION

Despite intensive efforts over the past 3 decades, a protective vaccine against human immunodeficiency virus type 1 (HIV-1), the causative agent of AIDS, is still wanting. This setback is partly explained by the formidable challenges posed by HIV-1, which has evolved a unique repertoire of immune-evasion mechanisms, most notably an extraordinary degree of antigenic variation, a widespread decoration of the exposed envelope (Env) surface with N-linked glycosylation, a marked inherent flexibility of the Env trimer, and a multistep entry process that allows the native Env spike to maintain key functional regions hidden from antibodies until reaching a sterically protected state proximal to the cellular membrane (1–5). In spite of these barriers, the past few years have witnessed major progress in our knowledge of the molecular and functional anatomy of the HIV-1 Env with the design and structural characterization of both soluble and membrane-bound native-like Env trimers (6–16), as well as with the cloning from infected individuals of broadly neutralizing antibodies (bNAbs) that target specific sites of HIV-1 vulnerability (1, 17, 18). High-resolution structures obtained by both X-ray crystallography and cryo-electron microscopy (cryo-EM) have permitted visualization of the prefusion state of the HIV-1 Env trimer, which adopts a closed, “stealth” conformation that limits exposure of vulnerable neutralization sites. Conversely, after CD4 binding, the trimer transitions toward a low-energy, open state characterized by a dramatic displacement of the variable loop complex at the trimer apex, formed by the coalescence of the first two (V1V2) and third (V3) variable loops of gp120 (1, 19–22). In this open conformation, the trimer is exposed to recognition by a wide variety of antibodies against sensitive regions such as the V3 tip and the so-called bridging sheet that provides the largest contact surface for coreceptor binding, which have otherwise little or no neutralizing activity because their target epitopes are largely concealed in the closed prefusion trimer (23–25).

The inherent conformational flexibility of the HIV-1 Env trimer and the dramatic alterations induced by CD4 binding represent important hurdles in the path toward the development of a protective vaccine. To overcome these obstacles, several groups have devised strategies to stabilize the trimer in its prefusion configuration as well as to reduce or abrogate its CD4-binding capacity. Thus, a precise definition of the structural mechanisms that stabilize the native trimer in its closed prefusion state is central for the design of effective vaccine immunogens. A series of recent observations have pointed to the trimer apex region as a global regulator of the open-closed state of the entire HIV-1 Env spike (4, 8, 26–31). Disruption of electrostatic or hydrophobic intramolecular interactions within this region was reported to reduce the trimer stability, leading to an altered neutralization profile, impaired infectivity, and/or increased gp120 shedding. We previously reported that two tyrosine residues in the V2 loop of gp120 (Y173 and Y177) can be posttranslationally modified by sulfation, which introduces a strong negative charge (26), and are critical to maintaining the trimer in a closed state by cementing the intramolecular interaction between V2 and the base of V3 (8). In separate studies, Herschhorn et al. identified hydrophobic residues in the V1V2 region, most notably L193 and, to a lesser extent, I154 and L175, that contribute to restraining the HIV-1 Env in state-1 conformation (27), while Bowder et al. found that three hydrophobic residues in the stem of the V3 loop, I307, I309, and F317, play a key role in preserving the gp120-trimer association and the overall trimer stability (4, 28–30). More recently, using *in silico* modeling, Da and Lin identified a large hydrophobic core at the trimer apex, flanked by the V1V2 and V3 loops and the β20/21 region of C4, that they predicted to play an essential role in regulating the closed trimer conformation and CD4-induced gp120 opening (31).

In this study, we aimed at dissecting the molecular anatomy of the HIV-1 Env trimer apex in order to identify structural constraints that regulate the global open/closed state of the trimeric spike and, thereby, modulate recognition by neutralizing antibodies. We utilized a combination of structural analysis and *in silico* free-energy calculations to predict the functional role of hydrophobic residues within the trimer apex, which aggregate into four hydrophobic clusters. These hydrophobic residues were individually mutated, and the resulting mutants were functionally characterized in order to validate the prediction and confirm their role in trimer stabilization. These results expand our knowledge of the structure-function relationships in the prefusion HIV-1 Env spike and may assist in structure-based vaccine design.

## RESULTS

### Four conserved hydrophobic clusters at the trimer apex are predicted to stabilize the prefusion HIV-1 Env spike.

**FIG 1:**
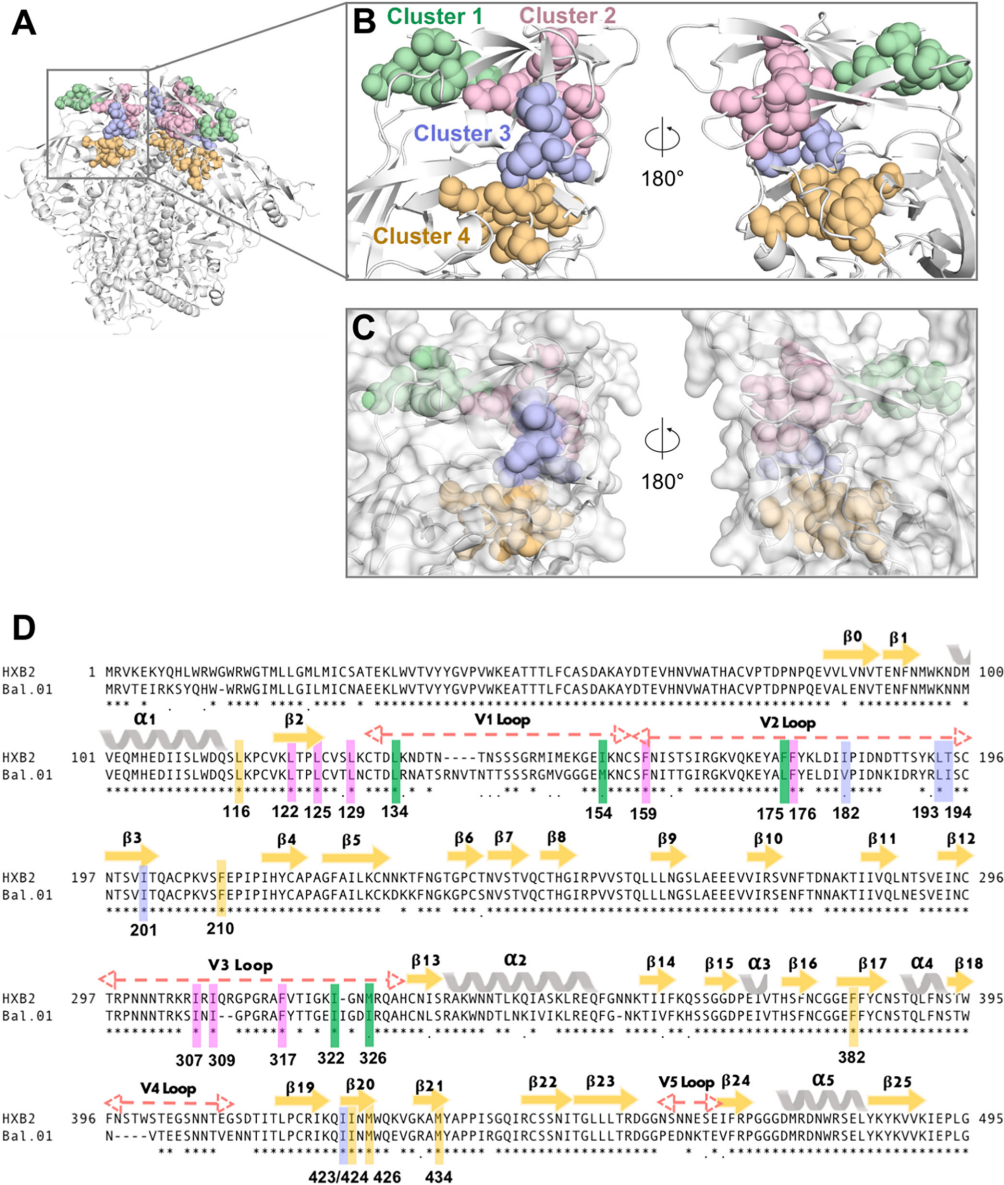
Visualization of four hydrophobic clusters in the apical region of the HIV-1 Env trimer. (A) Cartoon representation of the structure of the JR-FL SOSIP.664 trimer (PDB: 5FYK) with the four hydrophobic clusters highlighted in colors as indicated in panel B. (B) Close-up on the four apical hydrophobic clusters visualized in spheres (front and back views) and color coded: cluster 1 (green), cluster 2 (pink), cluster 3 (purple-blue), and cluster 4 (yellow-orange). (C) Surface representation view applied to the same structure (front and back) as in panel B. All the clusters are almost completely buried and inaccessible to the solvent in the prefusion trimer. (D) Amino acid sequence alignment of the BAL.01 and HXB2 gp120 glycoproteins with highlighted conserved residues in the four hydrophobic clusters (color coded): cluster 1 (green), cluster 2 (pink), cluster 3 (purple-blue), and cluster 4 (yellow-orange).

Analysis of multiple high-resolution structures of the HIV-1 Env trimer identified four conserved hydrophobic clusters that may play a major role in stabilizing the intraprotomer interactions between three overlying layers at the trimer apex represented by the V1V2 loop complex on the top, the V3 loop in an intermediate position, and the β20-21 strands within the C4 region of gp120 at the bottom (see Fig. S1 in the supplemental material). The location of the four hydrophobic clusters within the apical region of a single protomer of the JR-FL SOSIP.664 trimer (PDB ID: 5FYK) is presented in Fig. 1A with magnification in Fig. 1B. Surface representation shows that, in agreement with their hydrophobic nature, the four clusters are almost completely buried within the trimer apex (Fig. 1C). The four clusters contribute only 217.0, 81.6, 146.6, and 123.0 Å^2^, respectively, to the solvent-accessible surface in the JR-FL trimer crystal structure, with an average GXG tripeptide normalized surface area of 13.2% per cluster residue. Of note, none of these clusters appears to entertain energetically significant contacts with neighboring protomers, suggesting that their presumed stabilizing function occurs within the context of individual protomers. The position of the residues that form the four clusters in the amino acid (aa) sequence of gp120 is shown in Fig. 1D.

10.1128/mBio.00090-21.1 FIG S1 Layered composition of the apical region of the HIV-1 Env gp120 glycoprotein in the prefusion trimer. (A) Cartoon representation of the structure of the BG505 SOSIP.664 trimer (PDB: 5CEZ) with the three overlying layers that form the apical region highlighted by surface representation and different colors, as indicated. (B) The magnified boxes illustrate in greater detail the three-layered structure of the trimer apex with the V1V2 complex at the top, the V3 loop in an intermediate position, and the β20-21 strands in C4 at the bottom. The surface projection of the four hydrophobic clusters is grossly delineated by the ovals and colors (cluster 1, green; cluster 2, pink; cluster 3, light blue; cluster 4, yellow-orange). Download FIG S1, PDF file, 1.2 MB. Copyright © 2021 Zhang et al. 2021 Zhang et al. https://creativecommons.org/licenses/by/4.0/ This content is distributed under the terms of the Creative Commons Attribution 4.0 International license.

### (i) Cluster 1.

**FIG 2:**
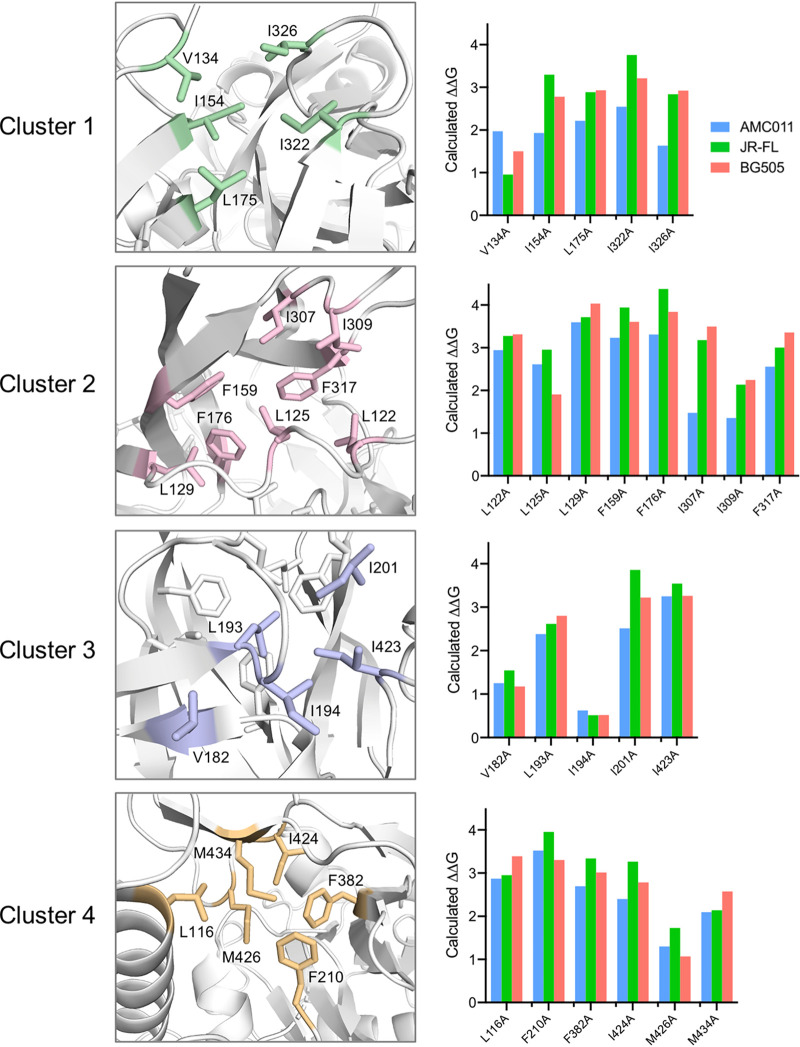
Structure of the four apical hydrophobic clusters and free-energy differences calculated for mutation of individual residues. Details of the hydrophobic interactions in each cluster (as indicated) with aa residues shown in stick representation (PDB: 5FYK). On the right side of each cluster structure, the bar plot shows the free-energy differences (ΔΔ*G*) calculated as described in Materials and Methods for alanine mutations introduced into the structural models of the AMC011, JR-FL, and BG505 SOSIP trimers. The indicated residues correspond to the JR-FL sequence (I154 is a leucine in BG505, while V134 is a leucine and I424 is a valine in AMC011).

The distalmost hydrophobic cluster includes two isoleucine residues on the V3 side, i.e., I322 and I326, and three residues on the V1V2 side, i.e., V134 in V1 and I154 and L175 in V2 (Fig. 2). The latter two aa positions were previously shown to play a role in Env stability (27). The two isoleucine residues in V3 and L175 in V2 are very highly conserved (>95%) among group M HIV-1 strains (>7,000 sequences in the Los Alamos database), while V134 and I154 are less conserved but almost invariably replaced by other hydrophobic amino acids (Fig. S2A).

10.1128/mBio.00090-21.2 FIG S2 Sequence conservation of the four apical hydrophobic clusters among global HIV-1 strains. (A) Sequence logo representation of aa residue conservation in hydrophobic cluster 1 (red frames) calculated from the sequence of 7,094 HIV-1 gp120 sequences deposited in the Los Alamos HIV-1 database. The degree of conservation is indicated by the height of the residue logo. (B) aa residue conservation in hydrophobic cluster 2. (C) aa residue conservation in hydrophobic cluster 3. (D) aa residue conservation in hydrophobic cluster 4. (E) Sequence logo representation of aa residue conservation in the four hydrophobic clusters based on HIV-1 neutralization tiers. Selected isolates categorized in the different tiers (tier 1, *n* = 23; tier 2, *n* = 61; tier 3, *n* = 17) were analyzed. Numbering and alignment are according to the HXB2 sequence. HIV-1 Env sequences were derived from the Los Alamos database, and the tier classification was derived from the work of Seaman et al. (M. S. Seaman, H. Janes, N. Hawkins, L. E. Grandpre, et al., J Virol 84:1439–1452, 2010, https://doi.org/10.1128/JVI.02108-09). Download FIG S2, PDF file, 0.8 MB. Copyright © 2021 Zhang et al. 2021 Zhang et al. https://creativecommons.org/licenses/by/4.0/ This content is distributed under the terms of the Creative Commons Attribution 4.0 International license.

### (ii) Cluster 2.

The second cluster encompasses three residues on the V3 side, i.e., I307 and I309 in the ascending limb of the loop and F317 in the descending limb, which were previously reported to promote trimer stability (28), along with five residues on the V1V2 side, i.e., L122, L125, and L129 in the V1V2 stem, F159 at the end of β-strand B in the V1V2 Greek key motif, and F176 at the end of β-strand C (Fig. 2). All V1V2 residues are almost universally conserved (>95%) among group M HIV-1 strains, while the V3 residues are highly conserved (>70%) and, when mutated, replaced by other hydrophobic amino acids (Fig. S2B).

### (iii) Cluster 3.

The third cluster encompasses four residues in the V1V2 stem, i.e., V182, L193, I194, and I201, and one isoleucine residue, I423, in the C4 region (Fig. 2). L193 was previously identified as a key residue for maintaining the trimer in the so-called state 1 (27). All cluster 3 residues are highly conserved among group M HIV-1 strains and, when mutated, replaced by other hydrophobic amino acids (Fig. S2C).

### (iv) Cluster 4.

The fourth cluster, most proximal to the gp120 core, encompasses one residue immediately upstream (i.e., L116) and one in the distal limb (i.e., F210) of the V1V2 stem, one residue in the C3 region, F382, and three residues in the C4 region, i.e., I424, M426, and M434 (Fig. 2). All the V1V2 residues are almost universally conserved (>95%) among group M HIV-1 strains, while the C4 residues are generally conserved and, when mutated, replaced by other hydrophobic amino acids (Fig. S2D).

Of note, when we compared the degree of conservation of aa residues within the four clusters among HIV-1 isolates categorized in different neutralization tiers (tier 1, 2, or 3) (32), we did not observe significant differences (Fig. S2E), suggesting that the apical hydrophobic constraints are not a primary determinant of neutralization tier classification.

### *In silico* energy calculations corroborate the stabilizing role of the four apical hydrophobic clusters.

To validate the role of the four hydrophobic clusters as stabilizing elements of the HIV-1 Env trimer apex, we performed an *in silico* assessment of the energetic cost of mutating each of the cluster residues to alanine using the Rosetta cartesian_ddg application (33). Figure 2 shows the calculated values associated with alanine substitutions at each position within each of the clusters starting from the experimental structures of three Env trimers selected to be representative of different clades and neutralization tiers: AMC011 (clade B, tier 1b; PDB ID: 6OLP), JR-FL (clade B, tier 2; PDB ID: 5FYK), and BG505 (clade A, tier 2; PDB ID: 6NNJ). All the alanine substitutions were predicted to have destabilizing effects, albeit to various extents, with the exception of I194A, which in all three backgrounds was predicted to be neutral, and V134A in the JR-FL background, which had a borderline calculated ΔΔ*G* of 0.96, near the threshold of 1.0 above which mutations were classified as destabilizing. Consistent with the high degree of conservation of the aa residues that form the four hydrophobic clusters (Fig. S2F), the calculated energetic effects of individual mutations were generally in agreement across the three Env backgrounds (Fig. 2). Thus, the energy cost associated with mutation of these residues does not seem to be significantly influenced by Env clades and neutralization tiers.

### Hydrophobic clusters 1 and 2 stabilize the apical variable loop complex.

Hydrophobic clusters 1 and 2 were predicted to stabilize the intraprotomer complex formed by coalescence of the V1V2 and V3 loops. We and others have reported that the tightness of the apical loop complex plays a critical role in maintaining the HIV-1 Env trimer structure in its closed, antibody-protected conformation (8, 26, 28). To evaluate the global impact of loosening the V1V2V3 loop complex on the HIV-1 Env spike structure and function, individual residues in clusters 1 and 2 were mutated to alanine in the backbone of a reference clade B Env (BaL, tier 1b), and infectious pseudoviruses bearing the mutated Envs were produced and tested for their neutralization profile. Two major classes of neutralizing reagents were employed: the first, targeting epitopes preferentially exposed in the open trimer conformation, included soluble CD4 (sCD4) and a panel of weakly/nonneutralizing monoclonal antibodies (MAbs) directed against the tip of the V3 loop (i.e., 447-52D), the coreceptor-binding site/bridging sheet (i.e., 17b, 412d), and the CD4-BS (i.e., F105); the second, targeting epitopes preferentially exposed in the closed trimer conformation, including a panel of potently neutralizing and trimer-preferring bNAbs directed against the V2-glycan supersite (i.e., PG9, PG16), the V3-glycan supersite (i.e., PGT128), the CD4-BS (i.e., VRC01), and the gp120-gp41 interface (i.e., PGT151, 35O22). The BaL Env was selected because it was extensively characterized in our previous study and shown to be highly sensitive to mutations that induce global conformational changes in the Env trimer (8).

**FIG 3:**
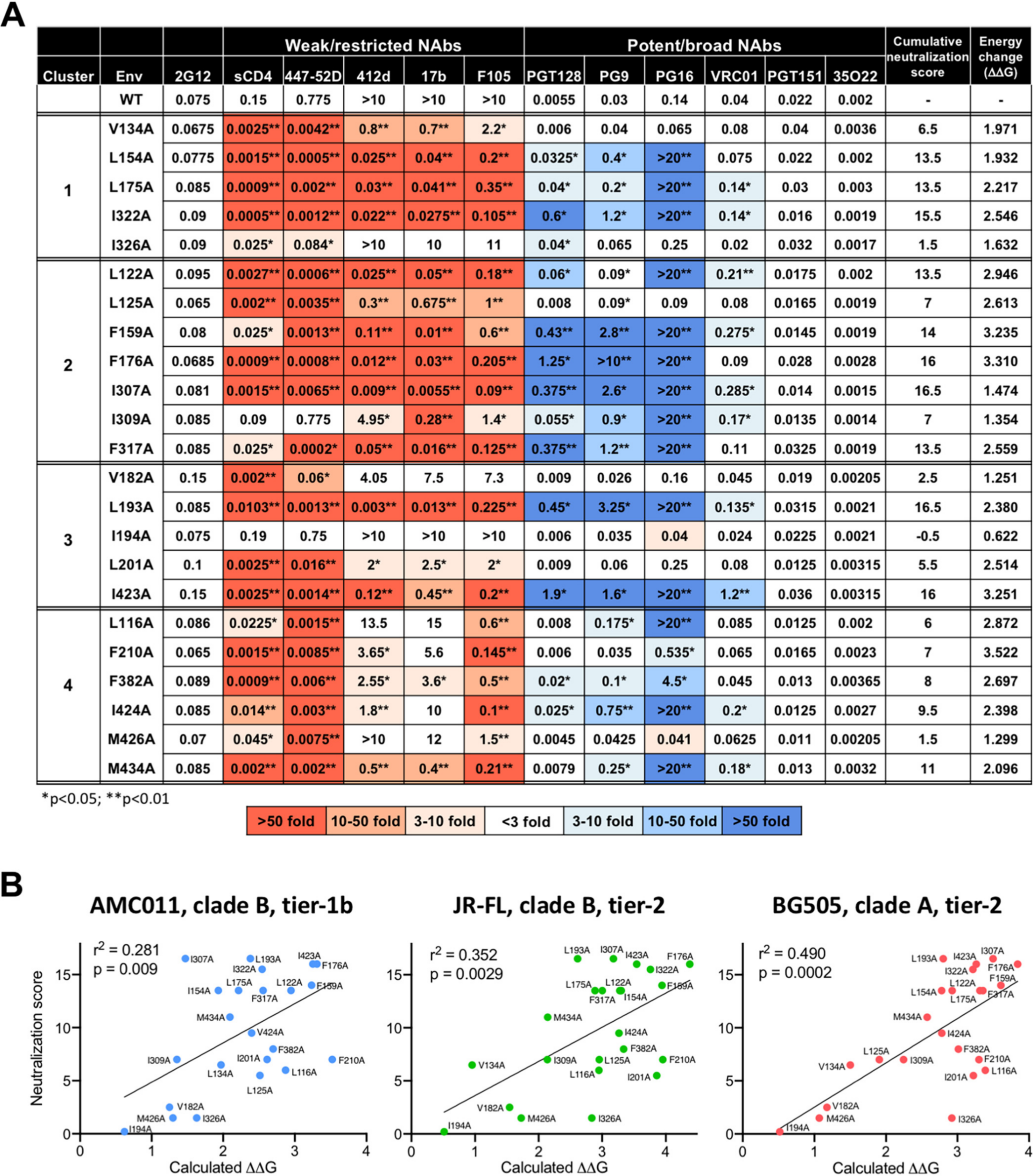
Effect of alanine substitutions in the four apical hydrophobic clusters on HIV-1 neutralization by sCD4 and monoclonal antibodies and correlation with energy scores. (A) Sensitivity to neutralization of HIV-1 BaL wild type and mutants bearing alanine substitutions in the four apical hydrophobic clusters. The values denote mean half-maximal inhibitory concentrations (IC_50_) expressed in micrograms from duplicate experiments performed on TZM-bl target cells. The color codes indicate the fold changes calculated by the ratio between the mean IC_50_ of each mutant and that of the wild-type (WT) Env, as specified in the legend at the bottom of the panel. Positive changes denote increased neutralization sensitivity (highlighted in different shades of red); negative changes denote decreased neutralization sensitivity (highlighted in different shades of blue). The asterisks indicate *P* values for the statistical comparison between neutralization of each mutant and the WT Env using an unpaired two-tailed *t* test (*, *P* < 0.05; **, *P* < 0.01). The trimer conformation-independent antibody 2G12 was used as a reference control. Mutant L129A (cluster 2) is not listed because it was not infectious in the TZM-bl assay. The cumulative neutralization score for each mutant was calculated based on the fold changes in neutralization with respect to the WT for each neutralizing reagent: a score of 2 was given for neutralization changes greater than 50-fold, a score of 1 for changes between 10- and 50-fold, a score of 0.5 for changes between 3- and 10-fold, and no score for changes below 3-fold. The energy values reported in the last column are those calculated for the AMC011 trimer (clade B, tier 1b). (B) Correlation between neutralization scores and *in silico* calculated ΔΔ*G* values for each mutant in three different HIV-1 Env trimer structures: AMC011, JR-FL, and BG505.

Most of the mutants in clusters 1 and 2 showed an altered neutralization profile characterized by divergent effects on the two classes of neutralizing reagents (Fig. 3A). On one side, the mutants exhibited an increased sensitivity to sCD4 and weakly/nonneutralizing MAbs specific for different sites (highlighted in different shades of red), while on the other they showed decreased sensitivity to trimer-preferring bNAbs specific for different sites (different shades of blue), a phenotype compatible with a generalized opening of the trimer structure. The only exceptions were the gp120/41 interface-specific bNAbs PGT151 and 35O22, whose neutralization potency was not affected by the mutations. Likewise, no significant impact was observed on 2G12, which was chosen as a control antibody because its binding is independent of the trimer conformational state (Fig. 3A). Of note, these dichotomous effects on the two classes of neutralizing reagents are identical to those previously reported for alanine substitutions of two tyrosine residues in the V2 region, Y173 and Y177, which play a key role in regulating the open/closed state of the HIV-1 Env spike (27). It is important to emphasize that, with the few exceptions noted below, the amino acid residues that form the hydrophobic clusters are not directly involved in the contact surfaces for the tested MAbs or for sCD4, which in many cases are located in distinct and distant domains of the gp120 glycoprotein. Thus, we can conclude that the observed effects on HIV-1 neutralization derived primarily from global allosteric changes in the Env trimer structure.

The cluster 1 mutations with the most dramatic impact on neutralization were L154A, L175A, and I322A, while V134A and I326A had a less marked effect with virtually no effect on trimer-preferring bNAbs (Fig. 3A). Among trimer-preferring bNAbs, PG16 was the most significantly and consistently affected, in agreement with its absolute trimer dependence, while PG9, PGT128, and VRC01 have different degrees of trimer preference, in this hierarchical order, but maintain the ability to bind monomeric gp120. The lack of impact on the interface-specific bNAbs 35O22 and PGT151, despite their trimer dependence, demonstrates that the apical hydrophobic constraints regulate only the distal part of the trimer, while leaving the membrane-proximal trimer stem unaltered. Similar results were previously obtained with V2 tyrosine mutants (27). The only residues in cluster 1 that are directly involved in an antibody epitope are I322 and I326 for PGT128 (PDB ID: 3TYG), which can explain the drastic loss of PGT128 activity observed against I322A; however, I322A had equally strong effects on virtually all neutralizing reagents tested, regardless of their target region (Fig. 3A), which corroborates a global alteration of the Env spike conformation. In contrast, the isolated loss of PGT128 function with mutant I326A, which otherwise had no effects on other trimer-preferring bNAbs, can be interpreted as a consequence of epitope disruption.

Cluster 2 is the most conspicuous of the four, being composed of 8 residues whose side chains converge to create a large hydrophobic cavity. Strikingly, all individual mutations in this cluster had significant effects on neutralization by both classes of antibodies. One mutant, L129A, completely lost infectivity, presumably due to catastrophic trimer destabilization, and could not be evaluated for neutralization. Among the other 7 mutations, the most consequential were L122A, F159A, F176A, I307A, and F317A, although two of them, F159A and F317A, showed a discrepancy between a strong effect on anti-CD4-BS MAbs like F105 and VRC01 and only a modest impact on sCD4, suggesting an incomplete opening of the trimer in this region (Fig. 3A). The remaining two mutations, L125A and I309A, selectively affected a single class of antibodies: L125A affected only weakly/nonneutralizing MAbs and sCD4, and I309A affected only trimer-preferring bNAbs, especially PG9 and PG16, in line with previous mutagenesis results (34). This behavior is suggestive of an asymmetrical opening of the trimer. Although two residues in cluster 2 participate in an antibody contact surface, i.e., Y317 for 447-52D (PDB ID: 4M1D) and L122 for 17b (PDB ID: 6CM3) and 412d (PDB ID: 2QAD), their mutation exerted broad effects on multiple neutralizing reagents of both classes, indicating a global opening of the Env spike. Altogether, the results obtained with cluster 1 and 2 mutants demonstrate that loosening of either of the two hydrophobic constraints that seal the intraprotomer V1V2-V3 interaction induces the Env trimer to adopt an open configuration, which is more accessible to sCD4 and weakly neutralizing antibodies but less efficiently recognized by trimer-preferring bNAbs, which depend on quaternary interaction with the closed trimer apex for binding.

### Hydrophobic clusters 3 and 4 anchor the apical variable loop complex to the gp120 core.

Hydrophobic clusters 3 and 4, which are located in a core-proximal position, were predicted to stabilize the interaction between the variable loop complex and the C4 region of gp120, thus tying the trimer apex onto the core of the glycoprotein. To evaluate the impact of loosening this interaction on the Env spike conformation, we tested the effects of individual alanine substitutions within these two clusters on neutralization using HIV-1 BaL Env-carrying pseudoviruses. As seen with clusters 1 and 2, alanine mutants in clusters 3 and 4 displayed variable degrees of alteration in their neutralization profile, again characterized by opposite effects on weakly/nonneutralizing reagents versus potently neutralizing trimer-preferring bNAbs (Fig. 3). Again, most of the residues in clusters 3 and 4 are not directly involved in the contact surface for the tested MAbs or sCD4.

In cluster 3, the mutations associated with the most dramatic changes in neutralization profiles were L193A and I423A, while V182A and I201A had lower effects limited to sCD4 and 447-52D and I194A showed a neutralization profile essentially identical to that of the wild type (WT) (Fig. 3). The dramatic loss of function seen with trimer-preferring bNAbs against L193A and I423A, two mutations in a core-proximal position at considerable distance from the V2-glycan and V3-glycan epitopes, reinforces the concept that global allosteric effects rather than epitope disruptions are responsible for the altered neutralization profiles. Again, no changes were seen with bNAbs 35O22 and PGT151. Of note, residue I423 is part of the binding epitope for both 17b (PDB ID: 6CM3) and 412d (PDB ID: 2QAD). Surprisingly, however, its mutation had only a modest effect on 17b neutralization.

Cluster 4 mutants had the most diverse behavior among the four clusters, exhibiting strong and consistent effects on neutralization by tip-targeting antibodies such as 447-52D, PG9, and PG16 but virtually no effect on coreceptor-binding site-directed antibodies. The most consequential mutations were F382A, I424A, and M434A, followed by L116 (primarily effective on 447-52D, PG9, and PG16) and F210A (mostly effective on sCD4, F105, and 447-52D), while M426A selectively increased the visibility of V3 (447-52D) but did not significantly affect any other region (Fig. 3). The negligible effect of the L116A mutation on neutralization by sCD4 may be due to a loosening of intracore C4 anchoring to the α1-helix in the C1 region, in line with the reported effects of inner-domain layer 1 mutations on the CD4-BS (35). Residue I424 is part of the contact surface for sCD4 (PDB ID: 1G9N) and F105 (PDB ID: 3HI1), which may explain the only limited increase in sCD4 sensitivity of mutant I424A, while the effect on F105 sensitivity was nevertheless remarkable. Finally, residue M434 is part of the binding epitopes for both 17b (PDB ID: 6CM3) and 412d (PDB ID: 2QAD), which may explain the only limited enhancement of neutralization by these MAbs on mutant M434A.

To confirm the role of the apical hydrophobic clusters in stabilizing the closed trimer configuration, we extended the analysis to another clade B HIV-1 strain, JR-CSF, with a tier 2 neutralization profile. Four mutants from cluster 2 and two from cluster 4, previously generated for a large mutagenesis study of the JR-CSF Env (36), were tested against the same panel of sCD4, weakly/nonneutralizing MAbs, and trimer-preferring bNAbs that was used for HIV-1 BaL. All the mutations in cluster 2 had dramatic and consistent effects on weakly/nonneutralizing antibodies and sCD4, as well as, with the exception of I309A, on trimer-preferring bNAbs; in contrast, cluster 4 mutants affected exclusively weakly/nonneutralizing antibodies and sCD4 (Fig. S3A). Despite the limited number of mutants tested, these results confirmed that apical hydrophobic clusters play a key role in stabilizing the closed trimer state also in a tier 2 strain.

10.1128/mBio.00090-21.3 FIG S3 Effect of individual alanine substitutions in apical hydrophobic clusters on HIV-1 JR-CSF neutralization. Sensitivity to neutralization of HIV-1 JR-CSF wild type (WT) and mutants bearing alanine substitutions in two apical hydrophobic clusters. (A) Sensitivity to sCD4 and a panel of human MAbs. The values denote mean half-maximal inhibitory concentrations (IC_50_) expressed in micrograms from duplicate experiments performed on TZM-bl target cells. The color codes indicate the fold changes (positive or negative) between the mean IC_50_ of each mutant and that of the WT Env, as specified in the legend at the bottom. The asterisks indicate *P* values for the statistical comparison between neutralization of each mutant and the WT Env using an unpaired two-tailed *t* test (*, *P* < 0.05; **, *P* < 0.01). The trimer conformation-independent antibody 2G12 was used as a reference control. The cumulative neutralization score for each mutant was calculated as described in the text and in the legend to Fig. 3. (B) Sensitivity of HIV-1 JR-CSF mutants to sera from HIV-1-infected patients with high or low neutralization potency. The mean IC_50_ for HIV-Ig on WT HIV-1 JR-CSF was >50 mg/ml; the mean half-maximal neutralization titers for the four patient sera were 1:3,600 for serum 18, 1:4,800 for serum 46, <1:20 for serum 22, and 1:40 for serum 31. Neutralization of mutants L122A and I424A was significantly increased (*P* < 0.01), compared to the WT, for low-neutralizing sera as determined using an unpaired two-tailed *t* test. Download FIG S3, PDF file, 0.1 MB. Copyright © 2021 Zhang et al. 2021 Zhang et al. https://creativecommons.org/licenses/by/4.0/ This content is distributed under the terms of the Creative Commons Attribution 4.0 International license.

### Neutralization profiles correlate with *in silico* energy calculations and infectivity.

Next, we correlated the experimentally observed effects of individual alanine substitutions on neutralization with the calculated energy values (ΔΔ*G*) in the context of the three HIV-1 Env trimer structures. A cumulative score system was devised to classify the neutralization profiles of each mutant based on the fold changes in neutralization (increase or decrease) observed with different reagents (Fig. 3A). As the two classes of neutralizing reagents used in this study are affected by trimer destabilization in opposite directions, the scores denote increased neutralization for weakly/nonneutralizing reagents and decreased neutralization for potent trimer-preferring bNAbs. A score of 2 was attributed to a >50-fold change in half-maximal inhibitory concentration (IC_50_) between the mutant and the WT Env, a score of 1 for a 10- to 50-fold change, a score of 0.5 for a 3- to 10-fold change, and no score for a <3-fold change. Although energy analysis was not performed on the BaL trimer due to the lack of a high-resolution structure, AMC011 provides the closest proxy as it belongs to the same clade (B) and tier phenotype (1b) and shares with BaL a high degree of sequence homology. Nevertheless, the correlation between neutralization scores and ΔΔ*G* was significant for all three trimer structures (Fig. 3B). Selected mutants (especially I326A and, to a lesser extent, L116A, L193A, and F210A) were distant from the best-fit line in all three structures due to a lower or higher neutralization score relative to their calculated energy, while mutant I307A was off the best-fit line only for the AMC011 trimer due to an underestimation of its energy role in the context of this structure. This appears to be related to discrepancies in the modeled backbone conformations between the AMC011 and JR-FL or BG505 trimer structures, which alter the environment about I307, resulting in a diminished calculated energy effect of mutating this residue to alanine (Fig. S4).

10.1128/mBio.00090-21.4 FIG S4 Structural explanation for anomalous behavior of I307 in the AMC011 Env ΔΔ*G* calculations. (A) Structural alignment of gp120 from the HIV-1 BG505 trimer (PDB ID: 6NNJ; light gray ribbon) to that of the JR-FL trimer (PDB ID: 5FYK; dark gray ribbon). Selected hydrophobic cluster 2 residues are shown in stick representation and colored in blue (6NNJ) or tan (5FYK). (B) Structural alignment of gp120 from the HIV-1 AMC011 trimer chain A (PDB ID: 6OLP; light gray ribbon) to that of the JR-FL trimer (PDB ID: 5FYK; dark gray ribbon). Selected hydrophobic cluster 2 residues are shown in stick representation and colored in green (6OLP) or tan (5FYK). (C) Structural alignment of HIV-1 AMC011 trimer chain C to JR-FL. Selected hydrophobic cluster 2 residues are colored in cyan (6OLP) or tan (5FYK). (D) Structural alignment of HIV-1 AMC011 trimer chain E to JR-FL. Selected hydrophobic cluster 2 residues are colored in pink (6OLP) or tan (5FYK). The altered orientations of I161 in 6OLP chains A and E and I309 in 6OLP chain C perturb the hydrophobic interactions about I307, which manifests in a diminished effect of mutating I307 to alanine for AMC011 in the ΔΔ*G* calculations performed in this study. Download FIG S4, PDF file, 0.8 MB. Copyright © 2021 Zhang et al. 2021 Zhang et al. https://creativecommons.org/licenses/by/4.0/ This content is distributed under the terms of the Creative Commons Attribution 4.0 International license.

Both the cumulative neutralization score and the calculated energy were then correlated with the pseudovirus infectivity of each mutant stock. Since HIV-1 infectivity is related to the structure of the prefusion Env spike, it was not surprising that trimer destabilization caused variable degrees of infectivity reduction (Fig. S5A). Such infectivity reduction was not correlated with the p24 content of each pseudovirus stock (Fig. S5B), indicating that mutated virions were inherently less infectious. One mutant, L129A, had no measurable infectivity even at the highest concentration in the TZM-bl assay and therefore could not be used for neutralization experiments. In contrast, all the other mutants could be evaluated by adjusting the input viral stock dilution to yield at least 10^5^ relative light units (RLU). For each mutant, we calculated the normalized infectivity index (Fig. S5C), i.e., the ratio between the reciprocal titers in the TZM-bl assay and the p24_Gag_ content of the pseudovirus stocks (i.e., reciprocal infectivity titer per pg of p24_Gag_). The infectivity index was significantly correlated with the neutralization score (Fig. S5D). Moreover, consistent with the correlation observed between neutralization score and change in free energy (ΔΔ*G*), the infectivity index was also correlated with the calculated free-energy difference for each alanine mutant (Fig. S5E). These results corroborate the concept that individual alanine mutations within the four apical hydrophobic clusters induce significant alterations in the trimer stability that, in turn, affect both the neutralization sensitivity and the infectivity of HIV-1.

10.1128/mBio.00090-21.5 FIG S5 Infectivity of hydrophobic cluster mutants and correlation with neutralization profile and calculated energy value. (A) Infectivity of pseudovirus stocks of HIV-1 BaL single alanine mutants as determined by limiting dilution using the TZM-bl assay. Mutations in each of the four hydrophobic clusters are highlighted by color codes: cluster 1, green; cluster 2, pink; cluster 3, blue; cluster 4, yellow-orange. (B) Correlation between infectivity and HIV-1 Gag p24 protein concentration in pseudovirus stock. The color codes are the same as in panel A. No correlation was found. (C) Normalized infectivity expressed as the ratio between reciprocal infectivity titers and HIV-1 Gag p24 protein concentration from each mutant. (D) Correlation between neutralization scores and normalized infectivity titer for HIV-1 BaL mutants. (E) Correlation between *in silico* calculated ΔΔ*G* values for alanine mutations introduced into the HIV-1 AMC011 Env trimer structure and normalized infectivity titer for HIV-1 BaL mutants. Download FIG S5, PDF file, 0.3 MB. Copyright © 2021 Zhang et al. 2021 Zhang et al. https://creativecommons.org/licenses/by/4.0/ This content is distributed under the terms of the Creative Commons Attribution 4.0 International license.

### Sera from HIV-1-infected patients contain high titers of antibodies that neutralize hydrophobic cluster mutants.

**FIG 4:**
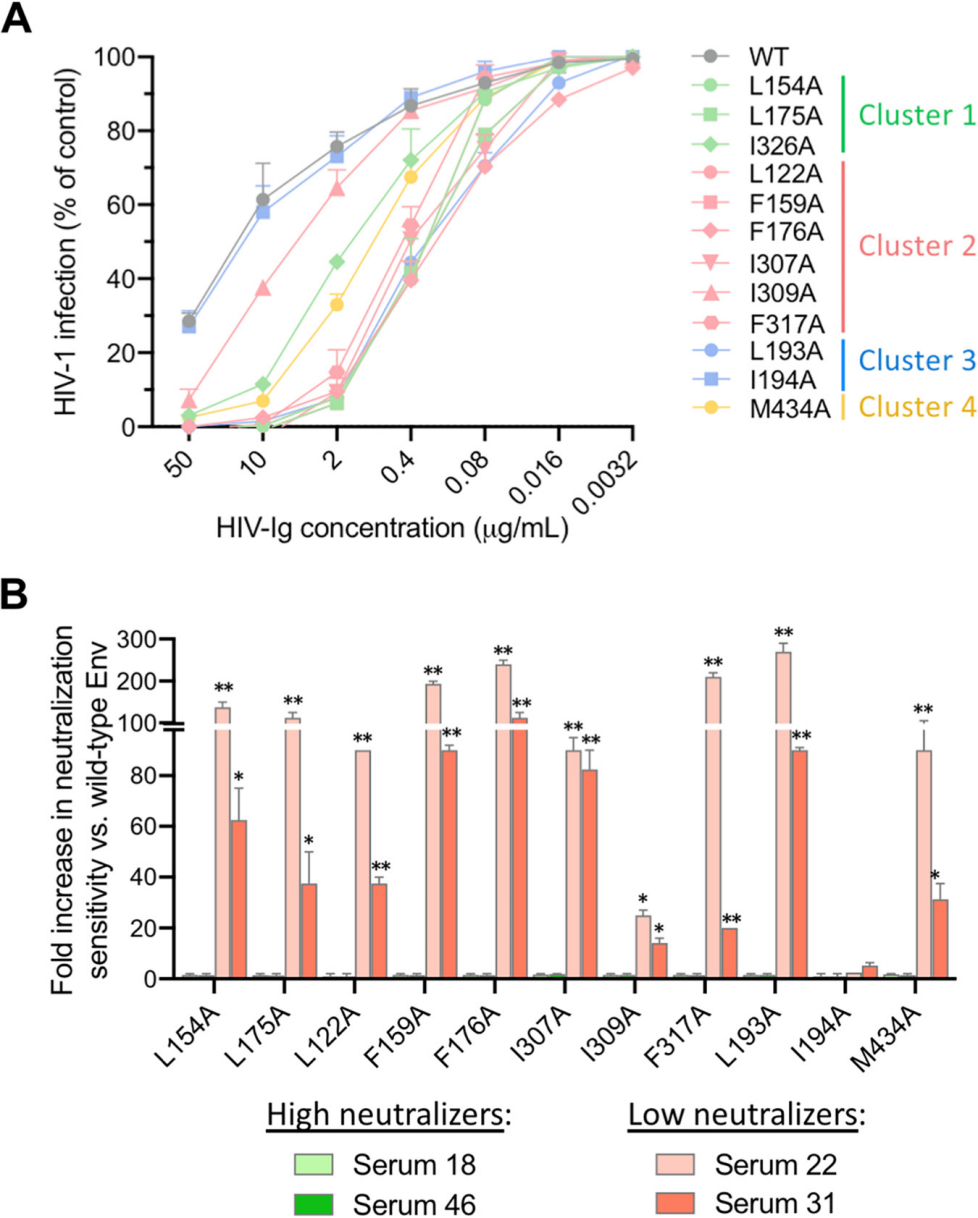
Effect of alanine substitutions in the four apical hydrophobic clusters on HIV-1 neutralization by sera from HIV-1-infected patients. (A) Sensitivity of HIV-1 BaL (tier 1b) mutants to neutralization by pooled immunoglobulins from chronically HIV-1-infected individuals (HIV-Ig). The mean half-maximal inhibitory concentrations (IC_50_) for HIV-Ig on HIV-1 BaL WT was 5 μg/ml. The neutralization tests were performed using the TZM-bl assay. The results obtained with all mutants except I309A and I194A were significantly different from those obtained with the WT Env, as determined by paired two-tailed *t* tests. (B) Neutralization sensitivity of HIV-1 BaL mutants to a panel of sera from HIV-1-infected patients with high or low neutralization potency. The mean half-maximal neutralization titers for the four patient sera against WT HIV-1 BaL were 1:7,500 for both serum 18 and serum 46, 1:20 for serum 22, and 1:100 for serum 31. The neutralization tests were performed using the TZM-bl assay. The results obtained with all mutants except I194A were significantly different from those obtained with the WT Env, as determined by paired two-tailed *t* tests.

Having established that mutations within the apical hydrophobic clusters alter the global HIV-1 Env configuration, resulting in a more open trimer configuration, we evaluated the sensitivity of selected mutants to neutralization by sera from chronically HIV-1-infected individuals. Initially, we tested a reference purified immunoglobulin preparation derived from pooled infected-patient sera (HIV-Ig), which has a moderate neutralizing activity and breadth (36). Selected HIV-1 BaL mutants (three from cluster 1, six from cluster 2, two from cluster 3, and one from cluster 4) were tested and compared with the WT Env. HIV-Ig had a significantly higher neutralization potency against all the mutants tested with the exception of I194A and I309A, with a distinct hierarchy of efficacy that overall mirrored the neutralization scores obtained with human MAbs (Fig. 4A).

Next, we tested the efficacy of four sera from chronically HIV-1-infected patients selected based on their neutralization potency and breadth: two strong neutralizers with high potency and breadth against tier 2 strains (sera 18 and 46) and two weak neutralizers with limited potency and breadth against tier 2 strains (sera 22 and 31). A striking discrepancy was observed between the two groups of sera. While strong neutralizers showed a similar potency against the WT and the mutants, weakly neutralizing sera displayed a dramatic and significant increase in neutralization capacity against all the mutants, with the sole exception of I194A, compared to the WT (Fig. 4B). Again, the hierarchy of neutralization gains for weakly neutralizing sera correlated well with the neutralization scores with human MAbs.

Both HIV-Ig and the representative patient sera were also tested against 3 mutants of the tier 2 HIV-1 Env, JR-CSF. Two mutants, L122A and I424A, showed a marked increase in sensitivity to neutralization by HIV-Ig and weakly neutralizing sera, while F210A, which had the lowest cumulative neutralization score, was equally insensitive to neutralization by all sera (Fig. S3B). As observed with HIV-1 BaL mutants, virtually no change in neutralization sensitivity was seen using potently neutralizing sera. Thus, despite the more protected configuration of the JR-CSF Env spike compared to that of BaL, these results confirmed the importance of the apical hydrophobic clusters as global regulators of the open-closed state of the trimer also in a tier 2 strain. Together, the data obtained with patient sera confirmed that HIV-1-infected individuals effectively mount robust antibody responses against the virus, but unfortunately the bulk of such antibodies have limited neutralizing capacity, being directed against concealed epitopes that are only partially or transiently exposed in the prefusion trimer.

## DISCUSSION

The present study was aimed at dissecting the functional anatomy of the HIV-1 Env trimer apex, a region that has emerged as a key regulator of the open/closed state of the viral Env spike and, therefore, of its vulnerability to neutralizing antibodies (8, 27, 28). The trimer apex is composed of three overlying layers within the gp120 subunit, i.e., the V1V2 loops at the top, the V3 loop in the middle, and the C4 β20-21 strands at the base, which form a tightly interconnected structural complex in the native prefusion spike. The integrity of this multilayered structure is essential to maintaining the entire Env trimer, not solely the apical region, in a closed, metastable conformation, which protects critical neutralization epitopes from the reach of host-produced antibodies. Binding to CD4 induces dramatic conformational changes in the Env spike that eventually result in the adoption of a low-energy postfusion (CD4-bound) state, in which multiple neutralization epitopes become fully accessible. During this process, the movable loop complex transitions from a fully folded centripetal position to an unfolded state in which the V1V2 and V3 loops diverge to eventually adopt opposite orientations. Such outward rotation of the loop structures may be likened to a lever mechanism that unlocks the entire Env trimer in order to trigger the conformational transitions required for viral entry. Importantly, the conformational changes that follow binding to CD4 constitute a multistep process whose discrete phases are still incompletely understood. Elucidating the structural events that take place along this ordered sequence may have fundamental implications not only for a better definition of the mechanics of HIV-1 entry but also for the design of effective inhibitors and vaccines.

Here, we used structural analysis combined with *in silico* energy calculations to predict the functional relevance of four major hydrophobic clusters located at the trimer apex in securing the tightness of this region, and we validated these predictions by mutating individual residues and evaluating the impact of mutagenesis on HIV-1 neutralization. Interestingly, in a recent *in silico* simulation using Markov state modeling applied to all-atom molecular dynamics simulations, Da and Lin reached similar conclusions by defining a 4-state transition pathway from closed to CD4-induced open conformations of gp120, positing that a hydrophobic core formed by many of the conserved apical residues that were mutated in our study plays a significant role in stabilization of the closed trimer (31). By loosening these structural constraints at the trimer apex through mutagenesis, we provided experimental evidence that partial opening of the trimer does not compromise the Env spike functional competence for receptor binding and viral entry. Only a single mutant, L129A, completely lost its functional competence and therefore could not be assessed for neutralization sensitivity in this study. Notably, however, all the mutants showed some degree of reduction in infectivity, despite a consistent increase in their capacity to interact with the CD4 receptor, which was significantly correlated with the impact of each mutation on the calculated free energy of folding (ΔΔ*G*) and, as a consequence, on neutralization sensitivity. This loss of replication fitness may be related, at least in part, to a reduced trimer association, as an impaired trimer stability was associated with increased levels of gp120 shedding (28). Although it remains a challenge to accurately predict the absolute change in free energy of folding associated with the introduction of a point mutation, the cartesian_ddg protocol has been shown to allow for a reasonable classification of mutations as destabilizing, stabilizing, or neutral and for rank-ordering mutations based on the magnitude of their effect (33, 37). Thus, the results of ΔΔ*G* calculations performed in the context of three different trimer structures were generally concordant, with small differences due to sequence heterogeneity, inherent conformational heterogeneity, model limitations related to the resolution of the experimental data, or, in the case of cluster 1 residues, variable interactions with modeled residues of the V1 loop between positions 134 and 154, which were not resolved in the three experimental structures. Most importantly, however, ΔΔ*G* values were found to be significantly correlated with the experimentally observed effects of the respective mutations on HIV-1 neutralization, reinforcing the concept that these residues are important mediators of trimer stability. The HIV-1 BaL strain was selected for the most extensive mutagenesis in this study because in a previous study we found that it was exquisitely sensitive to mutations that induce a global rearrangement of the trimer conformation. This may be partly related to its tier 1b neutralization profile, intermediate between the highly neutralization-sensitive tier 1a and the more resistant tier 2 phenotype. The intermediate tightness of the trimer assembly in the BaL Env provides an ideal model to identify even moderate contributions of individual residues to maintaining the trimer in closed configuration. In the absence of a high-resolution experimental structure of BaL Env for use in energy calculations, the clade B, tier 1b AMC011 Env offered a close proxy as it shares with BaL a high degree of homology. Furthermore, it must be emphasized that the calculated energetic costs accompanying mutation of the cluster residues to alanine were generally in agreement among the three trimers analyzed, which differed for clade and tier categorization, further corroborating the high degree of conservation of the apical hydrophobic constraints.

Strikingly, we found that even individual alanine substitutions within the four hydrophobic clusters at the trimer apex were sufficient to induce strong effects on the neutralization phenotype, which suggests that the HIV-1 Env exists in a precarious metastability, ready to snap into a more open conformation upon even minimal loosening of its structural constraints. Importantly, we confirmed the role of the apical hydrophobic clusters also in the context of a tier 2 HIV-1 strain, JR-CSF, despite its greater resistance to neutralization. These findings, together with the high degree of conservation of the four apical hydrophobic clusters across different HIV-1 clades and neutralization tiers, suggest that the apex-stabilizing function of these constraints remained conserved during the evolution of the global HIV-1 epidemic.

The results of the present study confirmed the dichotomous nature of neutralization epitopes present on the HIV-1 Env spike. We found that mutations within the four hydrophobic clusters increased the susceptibility to sCD4 and weakly/nonneutralizing antibodies and, at the same time, reduced the susceptibility to bNAbs directed against different sites of HIV-1 vulnerability. This opposite behavior reflects the differential dependence of these two classes of antibodies upon the open versus closed states of the trimer. Thus, sCD4 and weakly/nonneutralizing antibodies clash against the defensive shield of the Env spike; hence, their binding is significantly enhanced by a partial opening of the trimer. Conversely, bNAbs represent a rare breed of antibodies that have evolved unusual structural and functional features, e.g., deep reach through extralong CDR3 regions, direct glycan recognition, and/or quaternary interactions, to latch onto the highly defended “stealth” outer surface of the prefusion Env spike, and therefore possess an inherent trimer specificity, if not dependence. Of note, we did not observe any effect of the destabilizing mutations on two gp120-gp41 interface-specific bNAbs, 35O22 and PGT151, despite their trimer preference. This observation is important because it proves that the HIV-1 Env spike has the ability to loosen the tightness of its distal structure without compromising the trimer integrity, which is critical to preserve the Env function.

Only a limited fraction of infected individuals develop potent and broad-spectrum neutralization, and this achievement typically requires many months or even years of sustained viral antigenic stimulation (18). We previously documented the presence of high titers of neutralizing antibodies against partially open HIV-1 Env trimers in the serum of infected patients in spite of their poor neutralizing capacity against the WT virus (8). For the purpose of this study, we selected four reference sera from patients with different neutralizing potencies and tested them against mutants of the apical hydrophobic clusters. Our results reveal a striking contrast in that potently neutralizing sera showed virtually the same activity against the WT and mutated Envs, whereas weakly neutralizing sera showed a dramatic enhancement of neutralization against the hydrophobic cluster mutants, which suggests the presence of high titers of antibodies against hidden neutralization epitopes, most likely the same epitopes targeted by weakly/nonneutralizing MAbs (i.e., the V3-loop tip, the coreceptor-binding site, and the CD4-BS). These observations are in line with the results of Moody and colleagues, who demonstrated high titers of antibodies to the CD4-BS and the V3 loop with breadth for tier 1 and autologous tier 2 viruses, but not for heterologous tier 2 viruses, which may play a role in constraining the viral quasispecies to a neutralization-resistant phenotype *in vivo* (38). Interestingly, however, anti-V3 antibodies that are otherwise restricted to tier 1 neutralization were shown to neutralize a limited group of heterologous tier 2 isolates, which appear to feature an inherently more open trimer configuration (39).

The present study provides further evidence that the maintenance of a closed trimer configuration is a mechanism of HIV-1 immune evasion. Although the host immune system deploys a major effort in its attempt to neutralize the virus, such antibodies are largely ineffective, being unable to penetrate the tight defensive shield of the closed Env spike. Thus, the prevalent antibody response to the HIV-1 Env is skewed toward epitopes that are accessible only in the open trimer (38). These findings have obvious implications for HIV-1 vaccine development as they illustrate the challenges that the host immune system has to face to develop potent, broad-spectrum neutralization. There is little doubt that, in order to be effective, neutralizing antibodies must recognize the closed prefusion Env conformation. This necessary precondition can explain why immunization with monomeric gp120 or with open or partially unprotected trimers has consistently failed to induce bNAb responses (17, 18, 40, 41). Attempts to develop effective Env-based immunogens must therefore aim at presenting to the immune system native-like Env trimers preferentially stabilized in the prefusion state, which reduces the inherent structural flexibility that allows the trimer to continuously explore more open, vaccine-irrelevant conformations. However, it should not be ignored that some nonneutralizing antibodies can mediate alternative, yet potentially efficacious, effector functions, such as antibody-dependent cellular cytotoxicity (ADCC) (42, 43), which has been suggested as a protective mechanism in the RV144 vaccine trial (44).

In summary, our work provides new insights into the structural constraints that maintain the HIV-1 Env trimer in its closed, metastable state, endowing it with a formidable defensive shield against antibody neutralization. Deepening our knowledge of the functional anatomy of the HIV-1 Env spike and the structural transitions that accompany the multistep process of viral entry can open new avenues for the prevention and treatment of HIV/AIDS.

## MATERIALS AND METHODS

### *In silico* energy calculations.

*In silico* ΔΔ*G* calculations were performed using the Rosetta cartesian_ddg application (33). PDB files 5FYK (7), 6NNJ (45), and 6OLP (46) were used as starting models of Env for HIV-1 strains JR-FL, BG505, and AMC011, respectively. The PDB files were first stripped of all nonprotein components and chains other than those corresponding to the gp120 and gp41 subunits. The missing gp120 loops were then treated as follows: for BG505, residues 56 to 65, 133 to 154, and 458 to 464 were grafted from gp120 chains in PDB 5CEZ (13), 6MU7 (47), and 6UDJ (48), respectively, following structural alignment. All other missing gp120 loops were built with Prime (49) using the Crosslink Protein task within the BioLuminate modeling environment (50). Loops of fewer than 5 residues were built *de novo*, whereas missing sequences of five or more residues were filled in based on a loop lookup procedure using a curated PDB. Anchoring residues, including L134 from 6OLP chain A, were moved as necessary to accommodate the built loop. The missing gp41 sequence was not built. Sequences of starting models were those of the respective constructs used in structure determination; all mutations present in the experimental constructs were observed to be removed or oriented away from the described hydrophobic clusters.

Rosetta calculations were performed on trimeric Envs from each of the three strains. As a single Env protomer (i.e., single gp120 and associated truncated gp41 chain) is modeled in the crystallographic asymmetric unit of both 5FYK and 6NNJ, the JR-FL and BG505 trimers were generated through application of crystallographic symmetry operations following preparation of the respective monomers as described above. 6OLP, in contrast, is a cryo-EM structure with explicit coordinates for a complete Env trimer. All structures were first relaxed with restrained backbone and side chain coordinates using the ref2015_cart score function weights and a schedule of increasing weights for the repulsive term in accordance with recommendations (https://www.rosettacommons.org/docs/latest/cartesian-ddG). One hundred decoys were generated, and the five having the lowest Rosetta score were used as inputs to separate series of cartesian_ddg calculations (replicates), each consisting of 24 cartesian_ddg runs, one for each hydrophobic cluster position. The mutation being evaluated was introduced simultaneously into all three gp120 chains. For each calculation, 100 mutant and 100 wild-type decoys were generated. The ΔΔ*G* was then calculated as the difference between the mean score of the five lowest-energy mutant decoys and the mean score of the five lowest-energy wild-type decoys. The results reported are the averages over the five replicates for each mutant scaled by a factor of 0.34, which was determined to bring calculated values into better agreement with experimental values for a benchmark set of proteins (33), and divided by three to reflect the effect of introducing the mutation into a single gp120 protomer. Calculations were performed using Rosetta release 2019.47 on the NIH HPC Biowulf cluster.

### Solvent-accessible surface area calculations.

Solvent-accessible surface area calculations were performed with the program AREAIMOL from the CCP4 software suite (51) using PDB 5FYK after removal of all nonprotein atoms and chains other than those corresponding to JR-FL SOSIP.664 trimer and application of crystallographic symmetry operations to generate the intact trimer. A point density of 60 per square angstrom was used.

### Sequence conservation analysis.

Sequence alignment of gp120 protein segments containing hydrophobic regions from all the available group M HIV-1/SIVcpz isolates (including both subtypes A to K and recombinant forms) was obtained from the Los Alamos HIV database (http://www.hiv.lanl.gov). The sequence logo was created using WebLogo (http://weblogo.berkeley.edu).

### HIV-1 gp160 mutagenesis.

Primers for mutagenizing HIV-1 BaL were designed using the online program provided by Agilent (https://www.agilent.com/store/primerDesignProgram.jsp). The oligonucleotides were synthesized by Eurofins Genomics. The gp160 genes from different HIV-1 strains were mutagenized by site-directed mutagenesis using the QuikChange II site-directed mutagenesis kit (Agilent Technologies). The correct mutagenesis clones were expanded with the Qiagen Plasmid Mega kit. The JR-CSF mutants were produced for a previous study (52) and obtained through the Vaccine Research Center of the NIAID.

### Pseudovirus preparation and infectivity and neutralization assays.

HIV-1 pseudoparticles expressing the WT Env and respective mutants were cotransfected with an Env-deficient backbone plasmid (pSG3^Δenv^) into HEK293T cells (female human embryonic kidney cells, obtained from the ATCC). To produce pseudoviruses,1 μg of each Env-expressing plasmid and 1 μg of the backbone plasmid were mixed in Opti-MEM medium (Gibco), and 4 μl of LiFect293 transfection reagent was added at a 1:2 DNA-to-reagent ratio, followed by a 15-min incubation at room temperature (RT). DNA-LiFect293 transfection reagent complex was then added to 1.5 million cells in a 6-well plate and incubated overnight at 37°C. After replacing the culture medium with 2 ml of fresh 10% fetal bovine serum (FBS)-Dulbecco’s modified Eagle’s medium (DMEM), the cells were incubated for 48 h at 37°C in a humidified incubator, after which supernatants containing pseudoviruses were harvested by centrifugation. For single-round HIV-1 Env-pseudovirus infection of TZM-bl cells (female epithelial carcinoma cells, HeLa derivative, obtained from the AIDS Reagent Program), MAbs, sCD4, or heat-inactivated patient sera were serially diluted 3- or 5-fold and incubated with the pseudovirus for 30 min at room temperature, and then 10,000 TZM-bl cells were added to 96-well flat-bottom plates in 10% FBS-DMEM in a total volume of 200 μl per well. After 48 h, the medium was carefully removed, and the reporter gene (luciferase) expression was detected by adding 50 μl of luciferase assay reagent per well (Promega). The cell lysates were transferred to Lumino-plates and measured with a luminometer (PerkinElmer). Relative light units (RLU) were recorded, and the final values were normalized against the values obtained with the WT Env set at 100%. All the samples were tested in duplicate or triplicate wells. Calculation of half-maximal inhibitory concentrations (IC_50_) using a dose-response curve fit with a 5-parameter nonlinear function and two-tailed *t* tests were performed using GraphPad Prism 8 (Prism). All graphs were plotted using Prism 8.

### Patient sera and ethics guidelines.

Serum was obtained from chronically HIV-1-infected patients attending the NIAID AIDS Clinic for routine medical visits and laboratory testing. All patients gave written informed consent for donating blood products for research use and the study protocol (https://clinicaltrials.gov/ct2/show/NCT00039689?term=02-I-0202&rank=1) received approval by the NIH Institutional Review Board. All samples were coded, and no patient identifier was accessible to the laboratory personnel involved in this study. Both male and female patients were enrolled without discrimination. The sera were previously screened for neutralization potency against a reference panel of HIV-1 Envs. Two high/broad neutralizers and two low/restricted neutralizers were randomly selected for this study.

### Data availability.

All data are available in the figures of the paper. Primary data are also available upon request.
