# Supplementary material for: Functional Anatomy of the Trimer Apex Reveals Key Hydrophobic Constraints That Maintain the HIV-1 Envelope Spike in a Closed State
Source: mBio. 2021 Mar 30;12(2):e00090-21. doi: 10.1128/mBio.00090-21 (PMC8092198; doi:10.1128/mBio.00090-21)
Supplement: FIG S1 [file mBio.00090-21-sf001.pdf]

Figure S1

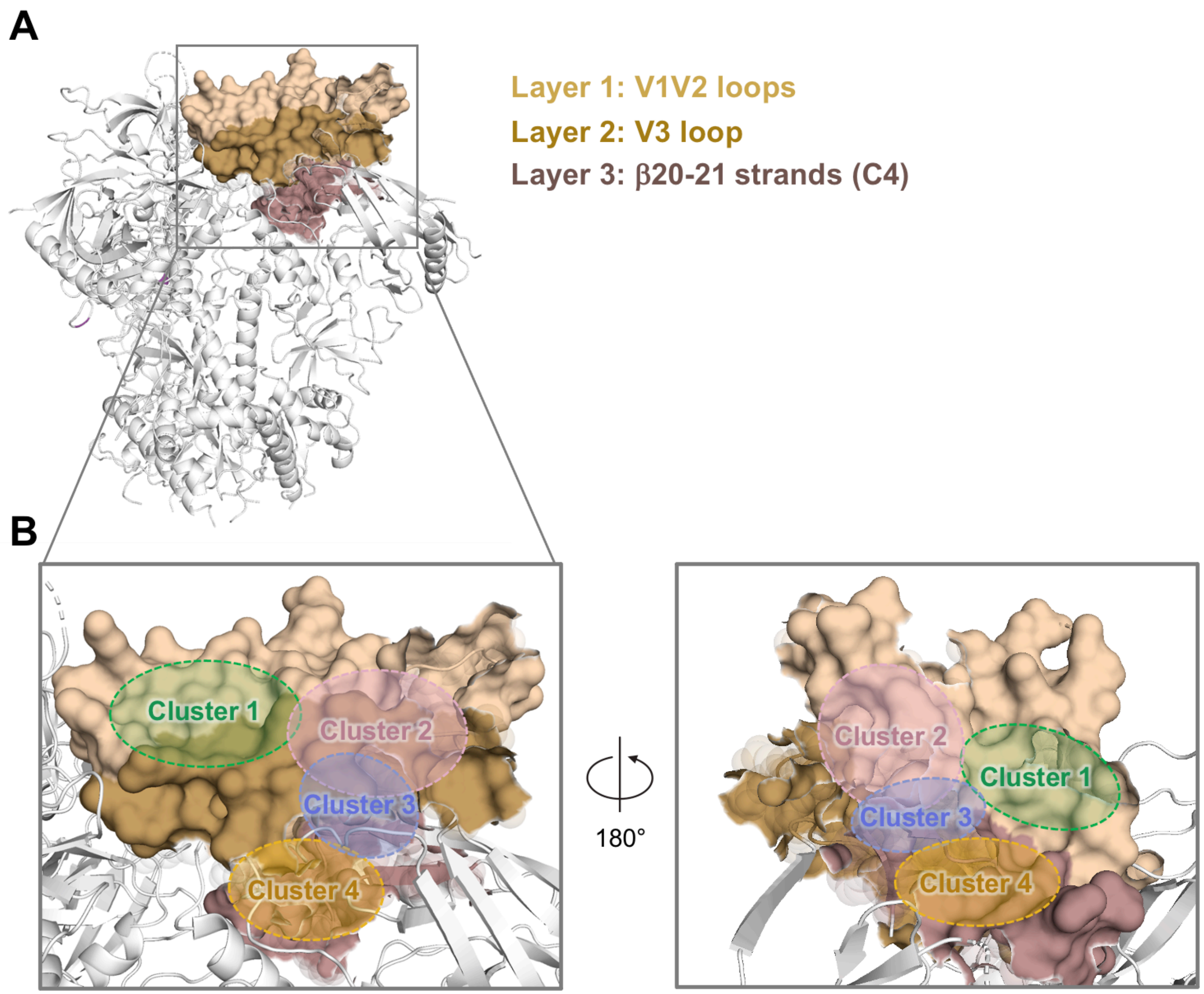

FIG S1 Layered composition of the apical region of the HIV-1 Env gp120 glycoprotein in the prefusion trimer. (A) Cartoon representation of the structure of the BG505 SOSIP.664 trimer (PDB: 5CEZ) with the three overlying layers that form the apical region highlighted by surface representation and different colors, as indicated. (B) The magnified boxes illustrate in greater detail the three-layered structure of the trimer apex with the V1V2 complex at the top, the V3 loop in an intermediate position and the β20-21 strands in C4 at the bottom. The surface projection of the four hydrophobic clusters is grossly delineated by the ovals and colors (cluster 1, green; cluster 2, pink; cluster 3, light blue; cluster 4, yellow-orange).
