# Supplementary material for: Functional Anatomy of the Trimer Apex Reveals Key Hydrophobic Constraints That Maintain the HIV-1 Envelope Spike in a Closed State
Source: mBio. 2021 Mar 30;12(2):e00090-21. doi: 10.1128/mBio.00090-21 (PMC8092198; doi:10.1128/mBio.00090-21)
Supplement: FIG S2 [file mBio.00090-21-sf002.pdf]

Figure S2

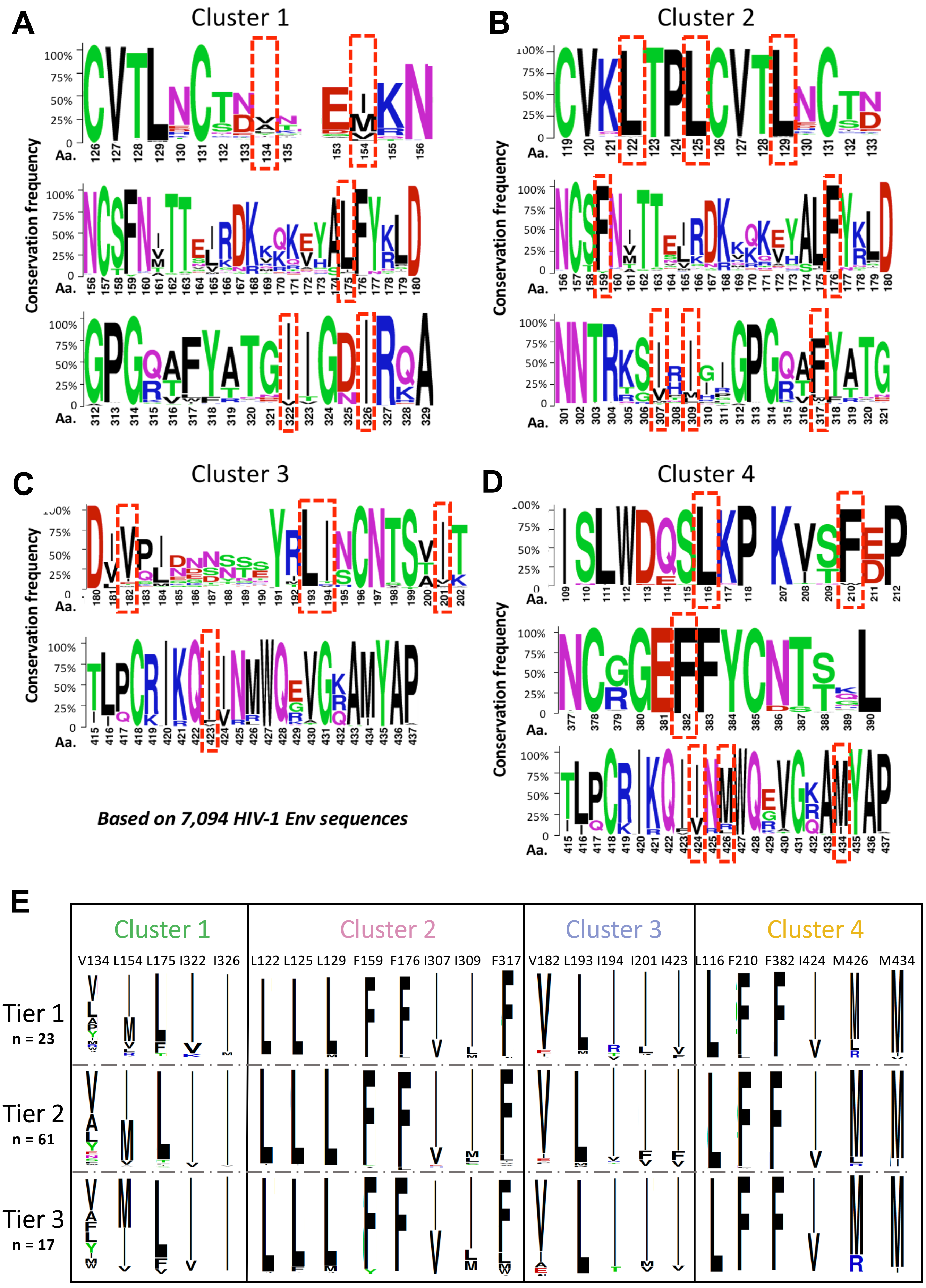
A
Cluster 1
B
Cluster 2
C
Cluster 3
D
Cluster 4
Conservation frequency
Aa.
100%
75%
50%
25%
0
Based on 7,094 HIV-1 Env sequences
E
Cluster 1
V134 L154 L175 I322 I326
Cluster 2
L122 L125 L129 F159 F176 I307 I309 F317
Cluster 3
V182 L193 I194 I201 I423
Cluster 4
L116 F210 F382 I424 M426 M434
Tier 1
n = 23
Tier 2
n = 61
Tier 3
n = 17

Figure S2 (legend)

FIG S2 Sequence conservation of the four apical hydrophobic clusters among global HIV-1 strains. (A) Sequence logo representation of aa. residue conservation in hydrophobic cluster 1 (red frames) calculated from the sequence of 7,094 HIV-1 gp120 sequences deposited in the Los Alamos HIV-1 database. The degree of conservation is indicated by the height of the residue logo. (B) Aa. residue conservation in hydrophobic cluster 2. (C) Aa. residue conservation in hydrophobic cluster 3. (D) Aa. residue conservation in hydrophobic cluster 4. (E) Sequence logo representation of aa. residue conservation in the four hydrophobic clusters based on HIV-1 neutralization tiers. Selected isolates categorized in the different tiers (tier-1, n = 23; tier-2, n = 61; tier-3, n = 17) were analyzed. Numbering and alignment are according to HXB2 numbering system. HIV-1 isolates Env sequences were derived from the Los Alamos database and the tier classification was derived from Seaman et al. [33].
