# Supplementary material for: Functional Anatomy of the Trimer Apex Reveals Key Hydrophobic Constraints That Maintain the HIV-1 Envelope Spike in a Closed State
Source: mBio. 2021 Mar 30;12(2):e00090-21. doi: 10.1128/mBio.00090-21 (PMC8092198; doi:10.1128/mBio.00090-21)
Supplement: FIG S3 [file mBio.00090-21-sf003.pdf]

Figure S3

A

Cluster	Env	2G12	Weak/restricted NAbs: sCD4	447-52D	412d	17b	F105	Potent/broad NAbs: PGT128	PG9	PG16	VRC01	PGT151	35O22	Cumulative neutralization score	Energy change (ΔΔG)
	WT	0.04	7	9.6	>10	>10	>10	0.001	0.004	0.002	0.15	0.0225	0.0825	-	-
2	L122A	0.045	0.005**	0.004**	0.05**	0.09**	0.44**	0.004*	0.2*	0.16*	0.46	nt	nt	13.5	3.279
	I307A	0.05	0.02**	0.01**	0.005**	0.005**	0.1**	0.03**	1*	0.5*	0.5*	0.016	0.085	15.5	3.178
	I309A	0.04	0.1**	0.1**	0.4**	0.1**	0.3**	0.001	0.009	0.015*	0.04	0.0215	0.09	10	2.138
	F317A	0.05	0.05**	0.04**	0.05**	0.01**	0.2**	0.001	0.01*	0.02*	0.02	0.014	0.08	11	3.003
4	F210A	0.05	0.8**	0.1**	>10	>10	>10	0.001	0.006	0.0016	0.25	0.0095	0.08	4	3.952
	I424A	0.033	0.04**	0.006**	3.6*	8	0.3**	0.0008	0.007	0.0015	0.22	0.016	0.11	6.5	3.266

*p<0.05; **p<0.01

>50 fold	10-50 fold	3-10 fold	<3 fold	-3-10 fold	-10-50 fold	>-50 fold

B

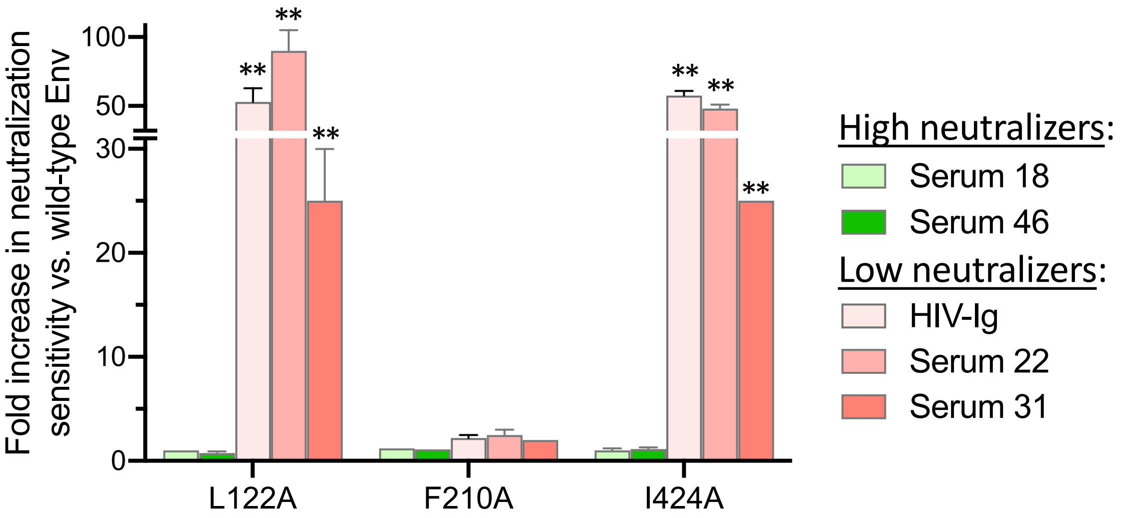

FIG S3 Effect of individual alanine substitutions in apical hydrophobic clusters on HIV-1 JR-CSF neutralization. Sensitivity to neutralization of HIV-1 JR-CSF wild-type (WT) and mutants bearing alanine substitutions in two apical hydrophobic clusters. (A) Sensitivity to sCD4 and a panel of human mAbs. The values denote mean half-maximal inhibitory concentrations (IC_{50}) expressed in micrograms from duplicate experiments performed on TZM-bl target cells. The color codes indicate the fold changes (positive or negative) between the mean IC_{50} of each mutant and that of the WT Env, as specified in the legend at the bottom. The asterisks indicate p values for the statistical comparison between neutralization of each mutant and the WT Env using an unpaired two-tailed t-test (* $p<0.05$; ** $p<0.01$). The trimer conformation-independent antibody 2G12 was used as a reference control. The cumulative neutralization score for each mutant was calculated as described in the manuscript and in the legend to Fig. 3. (B) Sensitivity of HIV-1 JR-CSF mutants to a sera from HIV-1 infected patients with high or low neutralization potency. The mean IC_{50} for HIV-Ig on WT HIV-1 JR-CSF was >50 μg/mL; the mean half-maximal neutralization titers for the four patient sera were: 1:3600 for serum 18; 1:4800 for serum 46; <1:20 for serum 22; and 1:40 for serum 31. Neutralization of mutants L122A and I424A was significantly increased ($p<0.01$), compared to the WT, for low neutralizing sera as determined using an unpaired two-tailed t-test.
