# Supplementary material for: Functional Anatomy of the Trimer Apex Reveals Key Hydrophobic Constraints That Maintain the HIV-1 Envelope Spike in a Closed State
Source: mBio. 2021 Mar 30;12(2):e00090-21. doi: 10.1128/mBio.00090-21 (PMC8092198; doi:10.1128/mBio.00090-21)
Supplement: FIG S4 [file mBio.00090-21-sf004.pdf]

Figure S4

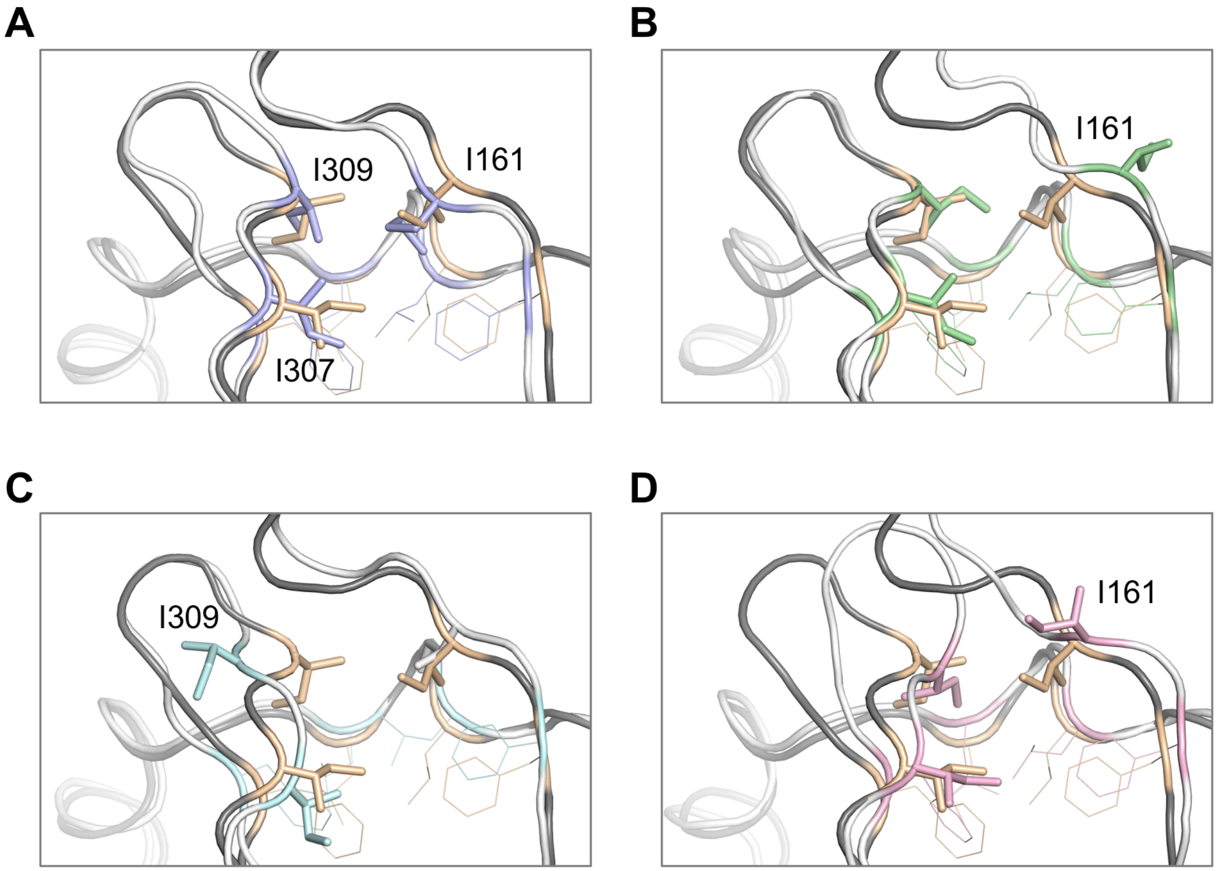

FIG S4 Structural explanation for anomalous behavior of I307 in the AMC011 Env ΔΔG calculations. (A) Structural alignment of gp120 from the HIV-1 BG505 trimer (PDB ID: 6NNJ; light grey ribbon) to that of the JR-FL trimer (PDB ID: 5FYK; dark grey, ribbon). Selected hydrophobic cluster-2 residues are shown in stick representation and colored in blue (6NNJ) or tan (5FYK). (B) Structural alignment of gp120 from the HIV-1 AMC011 trimer chain A (PDB ID: 6OLP; light grey ribbon) to that of the JR-FL trimer (PDB ID: 5FYK; dark grey, ribbon). Selected hydrophobic cluster-2 residues are shown in stick representation and colored in green (6OLP) or tan (5FYK). (C) Structural alignment of HIV-1 AMC011 trimer chain C to JR-FL. Selected hydrophobic cluster-2 residues are colored in cyan (6OLP) or tan (5FYK). (D) Structural alignment of HIV-1 AMC011 trimer chain E to JR-FL. Selected hydrophobic cluster-2 residues are colored in pink (6OLP) or tan (5FYK). The altered orientations of I161 in 6OLP chains A and E and I309 in 6OLP chain C perturb the hydrophobic interactions about I307, which manifests in a diminished effect of mutating I307 to alanine for AMC011 in the ΔΔG calculations performed in this study.
