# Supplementary material for: Functional Anatomy of the Trimer Apex Reveals Key Hydrophobic Constraints That Maintain the HIV-1 Envelope Spike in a Closed State
Source: mBio. 2021 Mar 30;12(2):e00090-21. doi: 10.1128/mBio.00090-21 (PMC8092198; doi:10.1128/mBio.00090-21)
Supplement: FIG S5 [file mBio.00090-21-sf005.pdf]

Figure S5

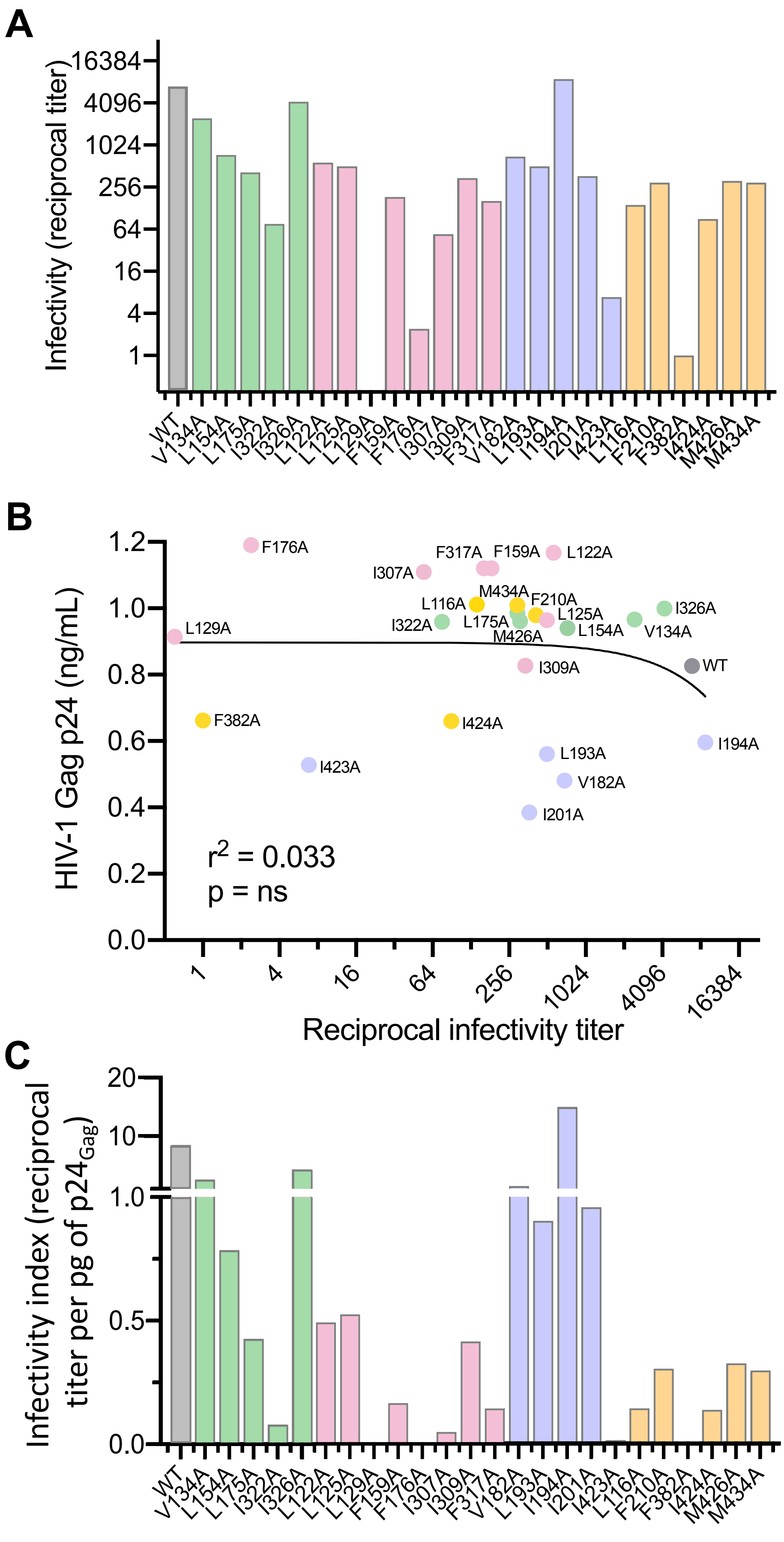
A
Infectivity (reciprocal titer)
16384
4096
1024
256
64
16
4
1
WT
V134A
L154A
L175A
I322A
I326A
L122A
L125A
L129A
F159A
F176A
I307A
I309A
F317A
V182A
L193A
I194A
I201A
I423A
L116A
F210A
F382A
I424A
M426A
M434A
B
HIV-1 Gag p24 (ng/mL)
Reciprocal infectivity titer
$r^2 = 0.033$
p = ns
F176A
I307A
F317A
F159A
L122A
L116A
M434A
F210A
I322A
L175A
L125A
M426A
L154A
V134A
I326A
L129A
WT
I309A
F382A
I424A
I194A
I423A
L193A
V182A
I201A
C
Infectivity index (reciprocal titer per pg of $p24_{Gag}$)

Figure S5 (continued)

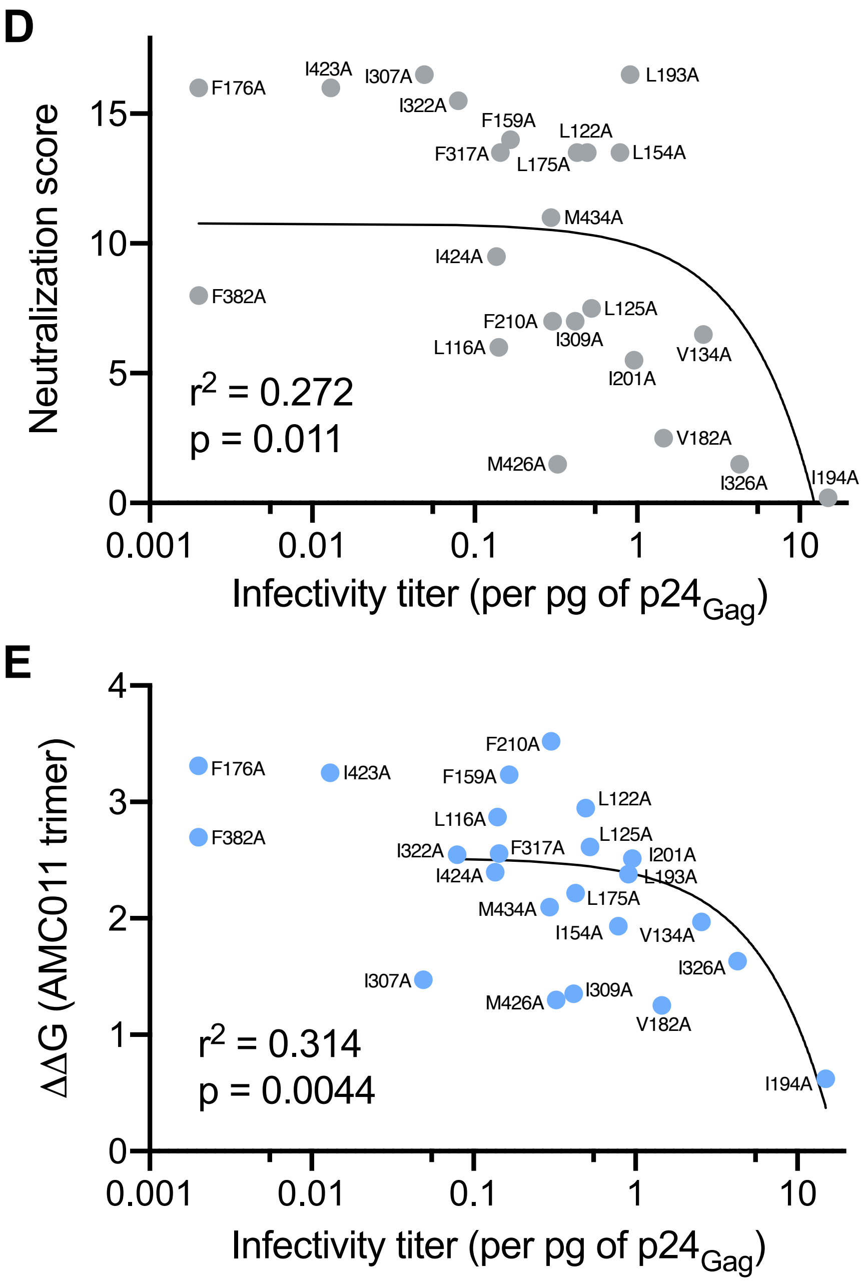

FIG S5 Infectivity of hydrophobic cluster mutants and correlation with neutralization profile and calculated energy value. (A) Infectivity of pseudovirus stocks of HIV-1 BaL single alanine mutants as determined by limiting dilution using the TZMbl assay. Mutations in each of the four hydrophobic clusters are highlighted by color codes: cluster 1, green; cluster 2, pink; cluster 3, blue; cluster 4, yellow-orange. (B) Correlation between infectivity and HIV-1 Gag p24 protein concentration in pseudovirus stock. The color codes are the same as in (A). No correlation was found. (C) Normalized infectivity expressed as the ratio between reciprocal infectivity titers and HIV-1 Gag p24 protein concentration from each mutant. (D) Correlation between neutralization scores and normalized infectivity titer for HIV-1 BaL mutants. (E) Correlation between *in silico* calculated ΔΔG values for alanine mutations introduced into the HIV-1 AMC011 Env trimer structure and normalized infectivity titer for HIV-1 BaL mutants.
